# Pneumococcal Vaccination Rates Among Patients Hospitalized for Pneumococcal Infection at a Community Teaching Hospital

**DOI:** 10.7759/cureus.90703

**Published:** 2025-08-21

**Authors:** Shikha Mishra, Hridya Harimohan, Kelly Ayabe, Royce Johnson, Michelle Fang

**Affiliations:** 1 Internal Medicine/Infectious Disease, Kern Medical, Bakersfield, USA; 2 Internal Medicine, Kern Medical, Bakersfield, USA

**Keywords:** infection, pneumococcal, streptococcus pneumoniae, underserved populations, vaccination

## Abstract

Background

Historically, lower pneumococcal vaccination (PV) coverage in minority populations and those with newly diagnosed chronic medical conditions, and increased hospitalizations for pneumococcal infections at Kern Medical (KM), a teaching hospital that serves as a safety net in Bakersfield, California, we sought to identify potential missed opportunities for pneumococcal vaccination at KM.

Methods

This quality improvement review was conducted to evaluate the PV status of patients who were hospitalized for pneumococcal disease from January 2023 through June 2024. Eligibility for PV was based on the Centers for Disease Control and Prevention (CDC)’s 2025 adult immunization schedule. PV history was identified through the California Immunization Registry. The primary endpoint was PV coverage in patients hospitalized for pneumococcal disease. Secondary endpoints included 14-day all-cause mortality, length of stay (LOS), and opportunities for vaccination at KM prior to admission.

Results

Thirty-five patients were hospitalized for pneumococcal disease at KM, including 18 cases of bacteremia. The mean age was 54 years, 77% were male, and 54% were Hispanic/Latino. Twenty-five patients met CDC criteria for PV; however, 92% of these patients were unvaccinated (68%) or undervaccinated (24%). Eight patients died within 14 days of positive culture, seven of whom were unvaccinated but eligible for PV. Mean LOS was 14 days with seven ICU days. Of the 23 unvaccinated or undervaccinated patients, the most common criteria met for PV included age (70%), chronic liver disease or alcoholism (35%), and diabetes mellitus (26%); only four patients between 50-64 years met criteria based on age alone. Of these, only five had primary care encounters at KM within one year of admission.

Discussion

Despite significant improvement in general incidence and outcomes of pneumococcal disease due to PV, vaccination rates still lag in minority communities and those with underlying medical conditions. The resultant and potentially avoidable impact on mortality and healthcare resources should serve as a call-to-action for public health and key stakeholders, including community pharmacies, to capture and augment opportunities for vaccination in these vulnerable populations.

Conclusion

This quality improvement review underscores significant gaps in pneumococcal vaccination coverage among adults hospitalized with pneumococcal disease at a safety-net hospital. Despite clear eligibility, the vast majority of the patients remained unvaccinated or undervaccinated prior to admission. However, achieving meaningful improvement in vaccine uptake will require a collaborative effort by public health agencies, hospital systems, and community partners to build trust, reduce vaccine hesitancy, and implement sustainable, equity-centered vaccination initiatives.

## Introduction

Pneumococcal disease remains one of the leading causes of preventable morbidity and mortality among adults in the United States. It encompasses a spectrum of clinical conditions, including pneumonia, bacteremia, and meningitis, all caused by *Streptococcus pneumoniae*. The burden is particularly high among older adults and individuals with chronic health conditions such as diabetes, chronic obstructive pulmonary disease (COPD), cardiovascular disease, and immunocompromising conditions [1]. Vaccination has proven to be an effective preventive measure against pneumococcal infections, with both the pneumococcal conjugate vaccine (PCV) and the pneumococcal polysaccharide vaccine (PPSV) showing reductions in invasive pneumococcal disease (IPD) and related hospitalizations [2,3]. 

In response to accumulating evidence of pneumococcal disease burden among younger adults with chronic medical conditions, the U.S. Centers for Disease Control and Prevention (CDC), based on recommendations by the Advisory Committee on Immunization Practices (ACIP), updated its recommendations in October 2024 to expand routine pneumococcal vaccination eligibility. This marked a significant shift, lowering the age of routine vaccination from 65 to 50 years and aligning adult immunization strategies more closely with the needs of medically vulnerable populations [4]. While this policy change has the potential to improve population health outcomes and reduce healthcare costs, its success hinges on effective implementation within clinical systems, particularly in under-resourced, high-risk communities. 

Historically, pneumococcal vaccine uptake among adults has been suboptimal, with pronounced disparities across racial, ethnic, and socioeconomic lines. National surveys have consistently shown that Black, Hispanic, and Native American adults have lower vaccination rates compared to non-Hispanic White adults [5,6]. Contributing factors include limited access to care, language barriers, mistrust in healthcare systems, lower health literacy, and inconsistent provider recommendations [7,8]. These disparities are exacerbated in safety-net healthcare systems, which serve a disproportionate number of patients from underserved backgrounds and may lack the infrastructure for comprehensive preventive care delivery. 

Kern Medical (KM) is a public safety-net teaching hospital located in Bakersfield, California, serving Kern County, a medically underserved area with significant health disparities and a predominantly Hispanic/Latino population. As of early 2024, an increase in hospitalizations for pneumococcal pneumonia and related complications was observed at KM, thus a quality improvement (QI) initiative was implemented to assess pneumococcal vaccination rates among these patients and identify potential missed opportunities to vaccinate them prior to onset of pneumococcal disease. This article was previously presented as a meeting abstract at the 2025 University of California, Los Angeles (UCLA)-Solomons conference 2025 on May 13, 2025.

## Materials and methods

As part of a broader quality improvement (QI) initiative, a retrospective chart review was conducted at Kern Medical (KM) to evaluate pneumococcal vaccination (PV) coverage among adult patients hospitalized with pneumococcal disease. The study period extended from January 1, 2023, to June 30, 2024. This initiative aimed to identify gaps in pneumococcal immunization and assess outcomes associated with vaccination status in this high-risk population.

Patients were eligible for inclusion if they were 18 years of age or older and had culture-confirmed pneumococcal disease that necessitated inpatient hospitalization. Only cases with microbiological evidence of *Streptococcus pneumoniae* from a sterile site were considered. Demographic and clinical data, including age, sex, comorbid conditions, and relevant hospitalization details, were extracted from the electronic health record (EHR). In addition to clinical information, outcome measures such as length of stay (LOS), mortality, and ICU admission were collected to evaluate patient trajectories during and after hospitalization.

Vaccination status was determined by reviewing documentation in both the EHR and the California Immunization Registry (CAIR), ensuring completeness and accuracy of immunization records. Patients were assessed for vaccine eligibility according to the 2025 CDC Adult Immunization Schedule, which incorporates age-based and risk-based criteria. Among those deemed eligible for pneumococcal vaccination, individuals with no documented history of receiving any pneumococcal vaccine were classified as unvaccinated. Patients with partial vaccination, such as those who initiated but did not complete the recommended series, or those overdue for an updated vaccine, were categorized as undervaccinated.

The primary outcome of the study was to determine pneumococcal vaccination coverage in patients hospitalized with pneumococcal disease. Secondary outcomes included 14-day all-cause mortality, hospital length of stay, and identification of missed opportunities for pneumococcal vaccination during healthcare encounters at KM prior to the index hospitalization. Missed opportunities were defined as previous interactions with the healthcare system where vaccination was indicated but not administered. Through this analysis, the study aimed to inform future strategies for improving adult immunization rates and reducing the burden of vaccine-preventable disease in hospitalized populations.

## Results

A total of 35 patients were hospitalized at KM with laboratory-confirmed pneumococcal disease between January 2023 and June 2024. Among these, 18 patients (51%) had bacteremia. The mean age of patients was 54 years (range: 50-72), with a predominance of male (77%) and Hispanic/Latino (54%) patients (Table 1).

**Table 1:** Baseline characteristics PV: Pneumococcal vaccination.

Characteristics N (%) or mean (range)	Total (n = 35)	Unvaccinated/ Undervaccinated (n = 25)	Vaccinated/ Not Eligible for PV (n = 10)
Age (years)	54 (22-91)	61 (42-91)	38 (22-63)
Male	27 (77)	19 (76)	8 (80)
Hispanic/Latino	19 (54)	15 (60)	4 (40)
Pneumococcal infection			
Bacteremia	18 (51)	14 (56)	4 (40)
Pneumonia only	17 (49)	11 (44)	6 (60)

**Figure 1:**
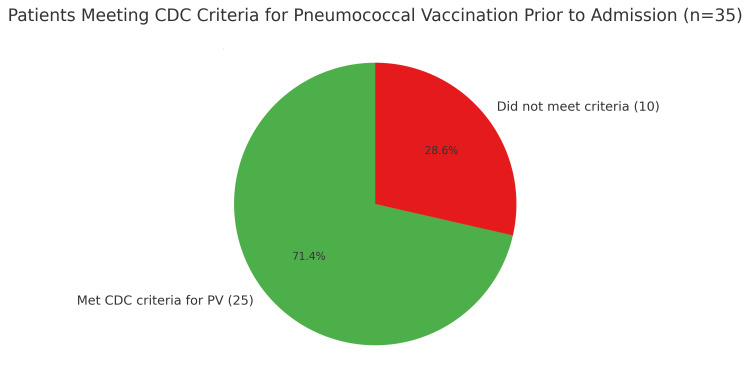
All patients hospitalized with pneumococcal disease

Of the 35 patients, 25 (71%) met CDC criteria for pneumococcal vaccination (PV) prior to admission (Figure 1). Eight patients died within 14 days of positive culture, seven of whom were unvaccinated but eligible for PV. Among the remaining eligible patients, 17 (68%) had no documented pneumococcal vaccination, and six (24%) were considered undervaccinated (i.e., had not completed the recommended vaccine series). 

**Figure 2:**
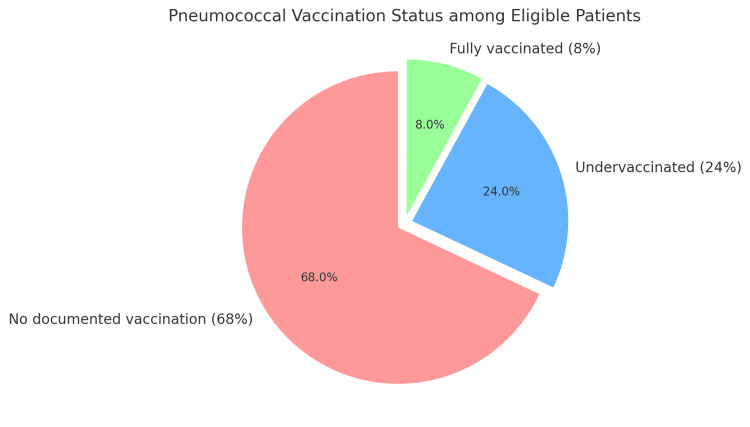
Patients eligible for pneumococcal vaccination

Clinical outcomes were notable for the death of eight patients (23%) within 14 days of initial positive culture for *S. pneumoniae*, all of whom were eligible for PV but had not received it (Figure 2). Hospital LOS was 14 days with an average of seven days spent in the intensive care unit (ICU), indicating substantial disease severity and healthcare resource utilization. 

**Figure 3:**
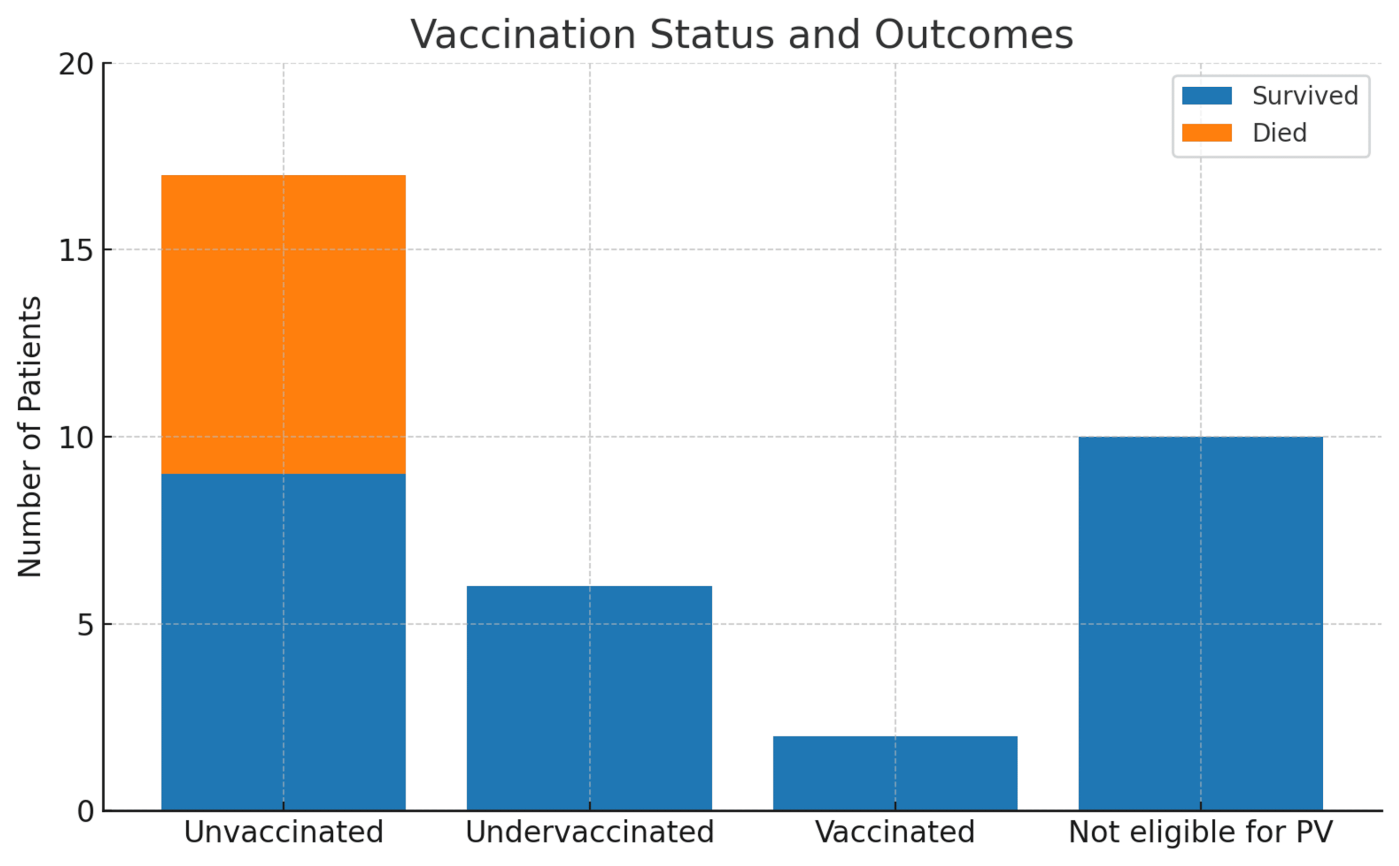
Clinical outcome- mortality at 14 days

Among the 23 unvaccinated or undervaccinated patients, the most common eligibility criteria met included age ≥ 50 years or chronic conditions such as diabetes mellitus and alcoholism/chronic liver disease (Figure 3). Only four patients between the ages of 50 and 64 met age-based criteria without additional comorbidities, reflecting the newly expanded CDC eligibility population. 

**Figure 4:**
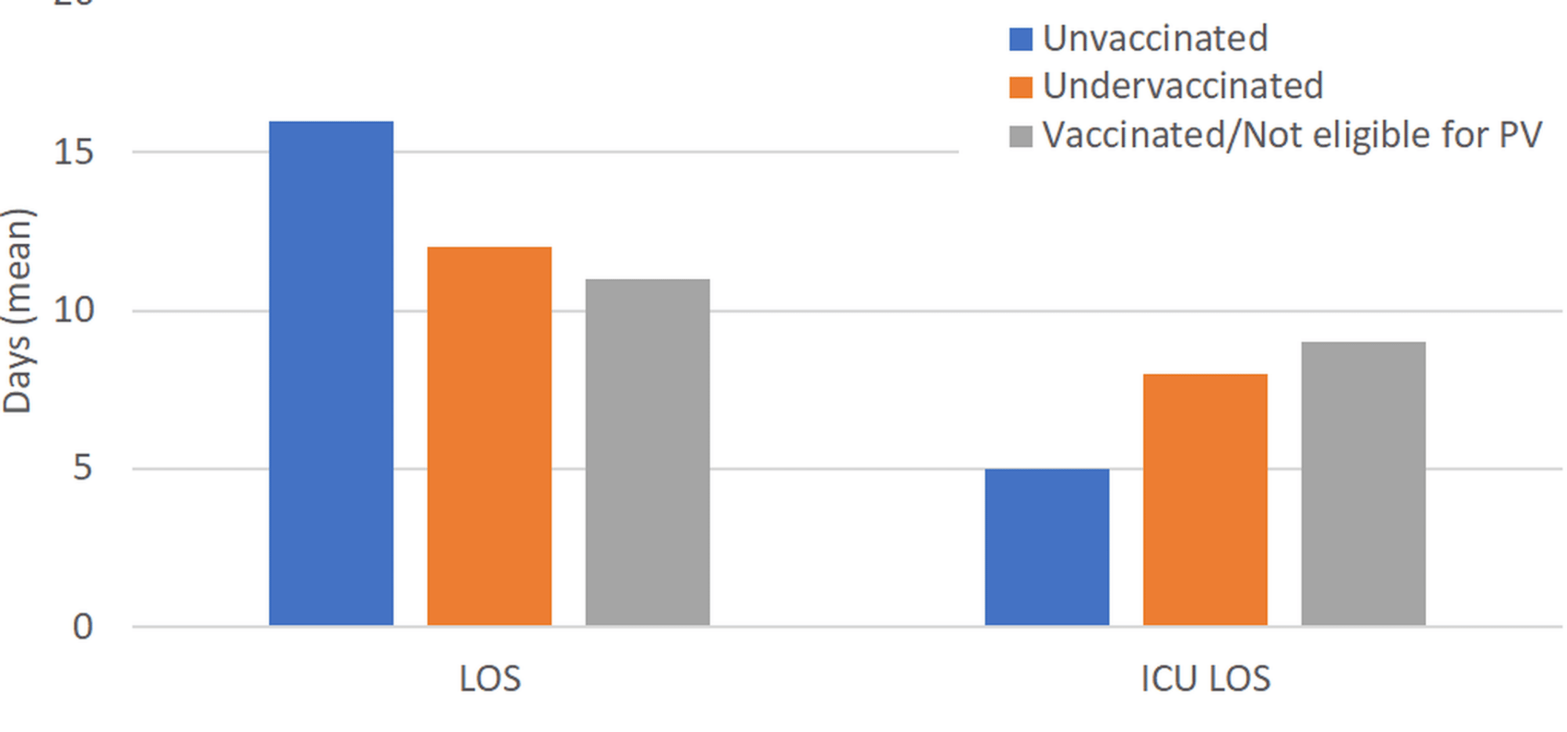
Clinical outcome- length of stay (overall and ICU)

**Figure 5:**
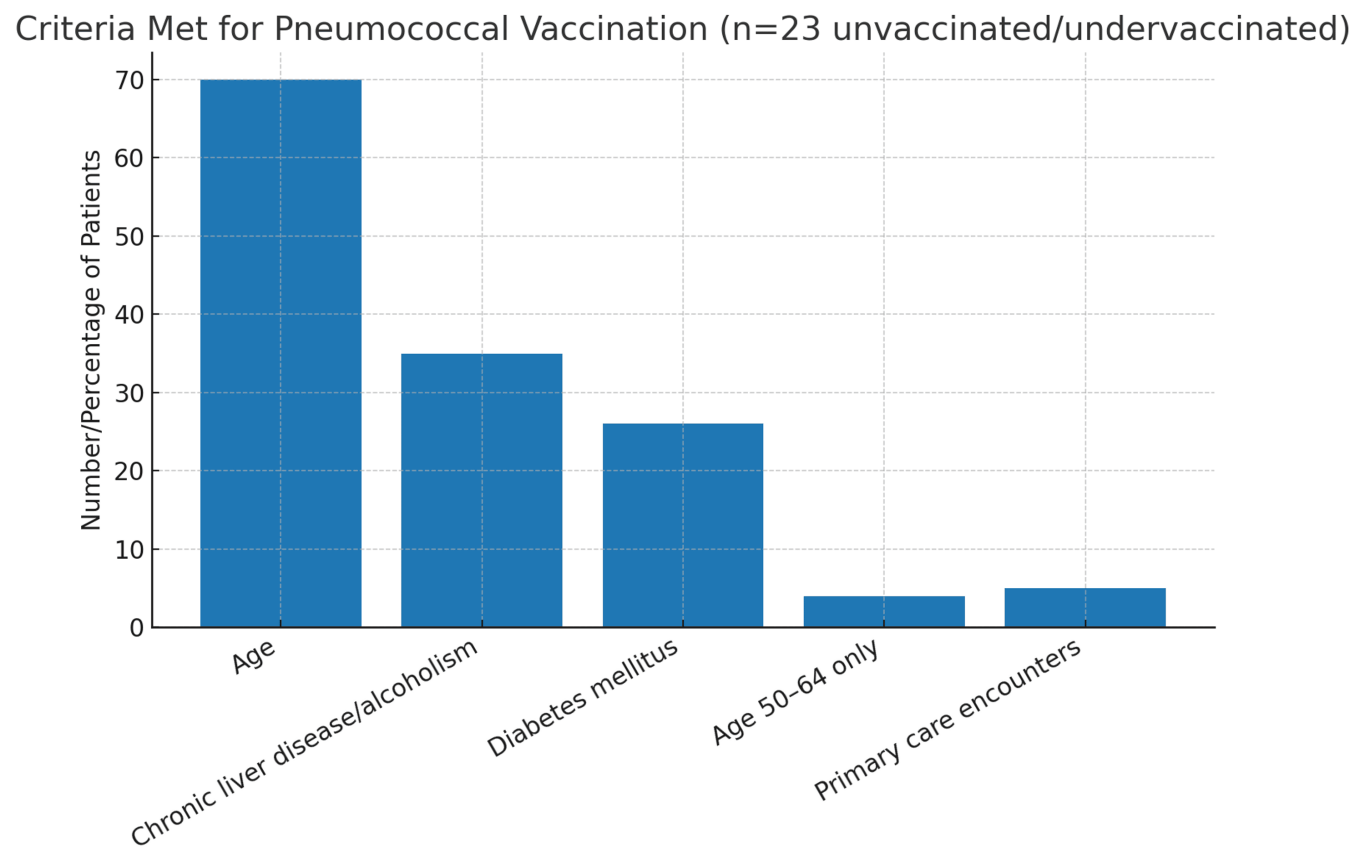
Criteria for pneumococcal vaccination eligibility

When examining care delivery opportunities, only five of the unvaccinated or undervaccinated patients had documented primary care visits at KM in the 12 months preceding hospitalization. None of the surviving patients were vaccinated during the index admission or scheduled for follow-up to do so as an outpatient (Figures 4, 5). 

## Discussion

Despite significant advances in the prevention of invasive pneumococcal disease (IPD) following widespread use of pneumococcal vaccines, vaccination coverage remains suboptimal among high-risk adults, particularly among racial and ethnic minorities, individuals with chronic health conditions, and those without consistent access to primary care [6]. In our cohort, 92% of eligible patients were either unvaccinated or undervaccinated at the time of their hospitalization for pneumococcal disease. This finding is consistent with national trends reported by Lu et al., who found that only 24.5% of adults aged 19-64 years with high-risk conditions had received a pneumococcal vaccine, with even lower rates among Hispanic and non-Hispanic Black patients [6]. Previous studies have estimated vaccine efficacy against invasive disease to be 60-70% in immunocompetent adults [7,8]. The observed 14-day all-cause mortality of 23% in this cohort, which only occurred in unvaccinated but vaccine-eligible individuals, underscores the continued morbidity and mortality burden of pneumococcal disease and the critical protective role of vaccination. 

The length of stay and ICU utilization were also substantial in our cohort, with an average hospitalization lasting 14 days, with an ICU duration of seven days. The shorter ICU LOS in the unvaccinated group was likely impacted by higher mortality rates in this group. Such findings translate into considerable resource use, reinforcing the economic rationale for increased vaccination outreach. Prior cost-effectiveness analyses have demonstrated that routine pneumococcal vaccination in adults with chronic conditions significantly reduces hospitalizations and ICU admissions, especially when implemented in populations served by safety-net hospitals [9]. 

One notable observation in our study was the low proportion (20%) of vaccine-eligible patients who had a primary care visit at Kern Medical within the year prior to hospitalization. This finding may suggest limited engagement with preventive care services and indicates a gap in both access and continuity of care. Given the evolving CDC recommendations, including the 2025 expansion of pneumococcal vaccination eligibility to adults aged 50-64 without additional risk factors, there is an urgent need to identify and engage patients across multiple healthcare touchpoints, not solely in primary care. Community pharmacies, mobile vaccine clinics, emergency departments, and specialty clinics offer practical venues to implement opportunistic vaccination strategies [10]. Addressing missed opportunities for pneumococcal vaccination at KM could not only align care delivery with national guidelines but also advance health equity, prevent avoidable hospitalizations, and enhance population-level preventive care. 

This review was limited by its retrospective nature and small sample size. Other factors not explored within this project include structural and systemic barriers such as limited insurance coverage, language and literacy challenges, vaccine hesitancy, and fragmented record-keeping, all of which compound the difficulty of ensuring equitable vaccine access. Integration of the California Immunization Registry (CAIR) into hospital EHR workflows and real-time vaccination alerts could facilitate timely administration, especially during inpatient admissions and emergency encounters. Additionally, staff education, standing orders, and nurse-led protocols have shown promise in improving inpatient immunization rates [11]. 

Ultimately, our findings support targeted interventions and proactive public health strategies to close the pneumococcal vaccination gap in underserved populations. Efforts must go beyond guideline dissemination to address the social determinants that drive low vaccine uptake. Health systems that serve socioeconomically vulnerable populations must take a leadership role in integrating vaccination opportunities into every point of care, incorporating strategies that target patients often missed by traditional models of care. Engagement with community pharmacies, Federally Qualified Health Centers (FQHCs), and culturally competent outreach programs can further bridge the gap for patients without regular access to primary care.

## Conclusions

This quality improvement review highlights concerning gaps in pneumococcal vaccination coverage among adults hospitalized with pneumococcal disease at a community-based safety-net hospital. Despite meeting clear eligibility criteria under the updated CDC guidelines, the majority of these patients had either not received any pneumococcal vaccination or were inadequately vaccinated prior to admission. These missed preventive opportunities were particularly striking given the associated clinical outcomes, namely, elevated 14-day mortality rates, extended hospital and ICU stays, and a notable lack of primary care engagement in the year preceding hospitalization. These patterns point to systemic vulnerabilities in both preventive care delivery and patient follow-up, particularly in populations already experiencing barriers to access.

In the context of the CDC’s recent expansion of routine pneumococcal vaccination recommendations to include younger age groups, these findings emphasize the urgency of re-evaluating current vaccine outreach and administration strategies. Hospitals and public health systems must seize this moment to design and deploy interventions that go beyond clinical settings. Improving vaccine uptake will require coordinated, multidisciplinary efforts among public health authorities, healthcare institutions, and community organizations. Such efforts should be anchored in building trust, addressing vaccine hesitancy through culturally sensitive education, and ensuring equitable access to vaccination, particularly in underserved and historically marginalized populations. Sustainable, equity-driven models of care are essential to bridge the gaps revealed in this review and to prevent avoidable morbidity and mortality from vaccine-preventable diseases.
